# Regional citrate anticoagulation with a substitute containing calcium for continuous hemofiltration in children

**DOI:** 10.1097/MD.0000000000017421

**Published:** 2019-10-04

**Authors:** Ke Bai, Chengjun Liu, Fang Zhou, Feng Xu, Hongxing Dang

**Affiliations:** Intensive Care Unit, Children's Hospital of Chongqing Medical University, Ministry of Education Key Laboratory of Children Development and Disorders, China International Science and Technology Cooperation Base of Child Development and Critical Disorders, Chongqing Key Laboratory of Pediatrics, Chongqing, P.R. China.

**Keywords:** anticoagulation, children, citrate, continuous hemofiltration

## Abstract

Regional citrate anticoagulation (RCA) was recommended as the first treatment option for adults by the Kidney Disease Improving Global Outcomes Kidney Foundation in 2012, for the characteristic of sufficient anticoagulation in vitro, but almost no anticoagulation in vivo. Traditionally, the substitute for RCA is calcium-free. This study investigated a simplified protocol of RCA for continuous hemofiltration (CHF) in children using a commercially available substitute containing calcium.

An analytical, observational, retrospective study assessed 59 pediatric patients with 106 sessions and 3580 hours of CHF. Values before and after treatment were compared, including Na^+^, ionic calcium (iCa^2+^) and HCO_3_^−^ concentrations, pH, and the ratio of total calcium to iCa^2+^ (T/iCa^2+^). In addition, in vivo and in vitro iCa^2+^, treatment time, sessions with continuous transmembrane pressure >200 mm Hg, and sessions with clotting and bleeding were recorded.

The average treatment time was 33.8 ± 10.1 hours. In vitro, 88.5% of iCa^2+^ achieved the target (0.25–0.35 mmol/L), and in vivo, 95.4% of iCa^2+^ achieved the target (1.0–1.35 mmol/L). There were 8 sessions with a transmembrane pressure >200 mm Hg and 3 sessions with filters clotted. After treatment, there were 2, 1, and 2 sessions with T/iCa^2+^ > 2.5 (implying citrate accumulation), iCa^2+^ < 0.9 mmol/L, and iCa^2+^ > 1.35 mmol/L. No sodium disorders were recorded. There were fewer cases of acidemia and more cases of alkalemia after treatment compared to before.

RCA-CHF with a substitute containing calcium and close monitoring could be a safe and effective treatment for children. In addition, the calcium test site in vitro and the adjustment of citrate should be given strict attention.

## 1 Introduction

Continuous renal replacement therapy (CRRT), as one of the three supportive technologies of critical illness, has been used widely to rescue critically ill children. As a kind of extracorporeal circulation technology, CRRT requires anticoagulation validly. The requirement for anticoagulation during CRRT is stricter for critically ill pediatric patients compared to stable or adult patients because of higher bleeding risk, longer CRRT duration, and slower blood flow.

Regional citrate anticoagulation (RCA) was recommended by the Kidney Disease Improving Global Outcomes Kidney (KDIGO) Foundation in 2012 as the first treatment option for adult patients with acute renal failure without contraindications.^[1]^ In RCA, anticoagulation occurs only in the extracorporeal circuit via the infusion of citrate, which chelates ionized calcium (iCa^2+^), resulting in very low Ca^2+^ levels in vitro. In addition, the levels of blood calcium and coagulation factors are restored after the blood is returned to the patient by supplying enough calcium.

In recent years, many studies have shown that RCA in CRRT could be used safely and effectively to treat children.^[2–11]^ However, this technique is complicated by the lack of commercially available physiological fluids, the various protocols that use different solutes and strategies, and the meticulous adjustment of citrate and calcium perfusions based on blood flow.^[2]^ Because 4% citrate solution contains high levels of calcium and sodium ions, the substitute should contain less sodium and less or no bicarbonate to avoid acid–base imbalances and hypernatremia. In addition, traditionally, the substitute for RCA had to be calcium-free. But, RCA reagents have not been commercially available in China. Efforts to prepare calcium-free substitutes have highly increased the risk of pollution, error, and blood infection, as well as the workload of nurses. Hence, the application of RCA has been severely restricted.

Port formula A solution (Terumo BCT Inc, Belgium) is widely used in China as a substitute for heparin anticoagulation. Commercialized hemofiltration basic solution (HBS) contains the same formula as port formula A and meets the basic requirements for RCA (less sodium and no bicarbonate), except that it contains calcium. Thus, this study investigated whether RCA for continuous hemofiltration (CHF) with a commercially available substitute solution containing calcium HBS and a simplified protocol in our hospital is safe and effective in children.

## 2 Methods

### 2.1 Study population

The study was approved by the ethical committee of Children's Hospital of Chongqing Medical University (File No. 2015.83). The study included 59 children who were admitted to our pediatric intensive care unit (PICU) at the Children's Hospital of Chongqing Medical University from September 2015 to July 2017. The inclusion criteria of the patients were as follows: all patients were critically ill; a course of RCA-CHF was administered; and the HBS was used as the substitute solution. Patients with hepatic failure or serum lactate >3.4 mmol/L were excluded from this study.

### 2.2 CHF protocol

The ACH-10 or Plasauto ∑ blood purification device (Asahi Kasei Kuraray Medical, Japan) was used for all patients. Filters (AEF-03, AEF-07, AEF-10) were selected according to patient body weight. Blood pathways were set up via arteriovenous connection, double-lumen central venous connection, or dual-channel venovenous modality. Venous or artery access was obtained by catheters, depending on the age and weight of the child.

Before treatment, the extracorporeal circulation tubes and filters were prerinsed with 3 L of saline containing heparin (40 mg/L), to be fully anticoagulated and to exhaust all air, and then with normal saline to drain the heparinized saline. If the volume of the extracorporeal circulation was >10% of the child's blood volume or the child had unstable hemodynamics, then erythrocyte suspension, plasma, or albumin was supplemented. Commercially available HBS (approval number: H20080452; Chengdu Qingshan Likang Pharmaceutical, China) was used as the substitute fluid (Na^+^ 113 mmol/L, iCa^2+^ 1.6 mmol/L, Mg^2+^ 0.797 mmol/L, Cl^−^ 118 mmol/L, and anhydrous glucose 10.6 mmol/L).

The citrate sodium anticoagulant (4%; approval number: H20058913; Sichuan Nightingale Biological, China) was pumped into the extracorporeal blood circulation by infusion pump (Optima PT, Fresenius SE & Co. KGaA, Germany) through a T-junction connected at the primer of the artery pipeline. Calcium gluconate (10%) was pumped into the extracorporeal blood circulation by an Agilia infusion micropump (Fresenius SE & Co. KGaA, Germany) through a T-junction connected at the end of the vein pipeline. If the double-lumen central venous catheter was used both for blood taking and returning, then the calcium gluconate was pumped into the body through other venous lines.

The initial parameters were set and simplified by the following formula^[5]^: blood flow rate = 3–5 (mL/kg/minute); displacement solution rate (mL/hour) = 35 mL/kg; citrate 4% rate (mL/hour) = blood flow rate (mL/minute) × (1.5–2); sodium bicarbonate 5% rate (mL/hour) = displacement solution (mL/hour)/(80–100); and calcium gluconate (10%) rate (mL/hour) = blood flow rate (mL/minute) × (0.1–0.2). However, from August 2015, the initial parameters were slightly modified as follows: blood flow rate (mL/minute) = 35 (mL/kg/minute); displacement solution rate (mL/hour) = 35 mL/kg; citrate 4% rate (mL/hour) = blood flow rate (mL/minute) × 1.8; sodium bicarbonate 5% rate (mL/hour) = displacement solution (mL/hour)/100; and calcium gluconate (10%) rate (mL/hour) = blood flow rate (mL/minute) × 0.1. For example, for a child weighing 10 kg, the initial parameters would be calculated as follows: blood flow rate = 5 × 10 = 50 mL/minute; displacement solution rate (mL/hour) = 35 × 10 = 350 (mL/hour); citrate (4% citrate) rate (mL/hour) = 50 × 1.8 = 90 mL/hour; and sodium bicarbonate 5% rate (mL/hour) = 350/100 = 3.5 (mL/hour).

**Figure 1:**
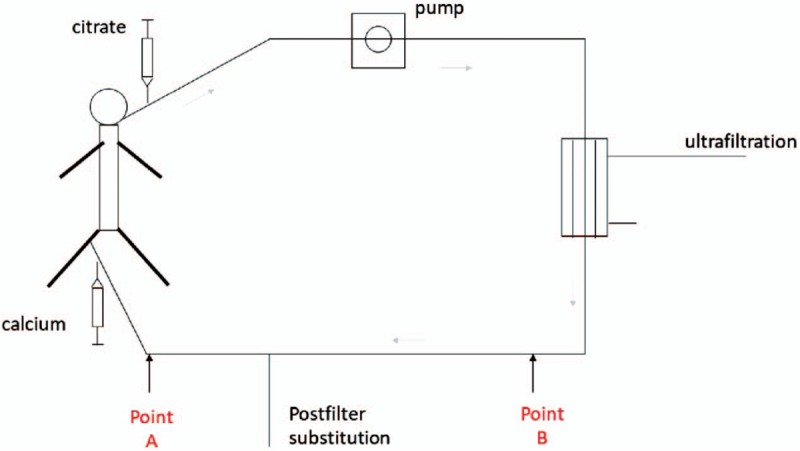
Regional citrate anticoagulation (RCA) schematic. Inhibition of clotting is limited only by the extracorporeal circuit. Citrate is infused into blood entering the extracorporeal circuit at a site proximal to the hemofilter. Citrate chelates calcium, thus making it biologically unavailable, and inhibits the propagation of a coagulation cascade. The extracorporeal circuit will not clot if the calcium ion concentration is maintained at 0.25 to 0.35 mmol/L. When using a calcium-free substitute, the calcium ion concentration is monitored at point B. However, when using a substitute containing calcium, we should detect the calcium ion concentration at point B, because the concentration of calcium ions will higher at point A compared to B.

First, the flow rate of the 4% sodium citrate was adjusted to maintain the in vitro ionized calcium concentration (iCa^2+^E), targeted 0.25 to 0.35 mmol/L, and was tested in the extracorporeal circulation pipeline at the site after infusion of the postfilter substitute (Fig. 1, point A), and was adjusted to maintain the targeted range (Table 1). Specifically, the flow rate of 4% sodium citrate was increased by 10% if the iCa^2+^E was <0.25 mmol/L, or was reduced by 10% if the iCa^2+^E was >0.35 mmol/L.

**Table 1:**
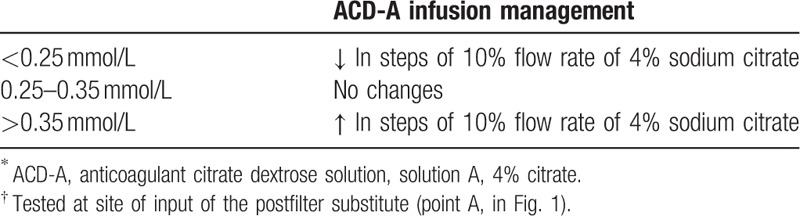
Management of ACD-A-infused solution^∗^ dependent on postfilter blood ionized calcium levels (iCa^2+^E)^†^.

Second, the infusion rate of 10% calcium gluconate was adjusted to maintain the patient's systemic blood ionized calcium concentration (iCa^2+^I) within the physiological range (1.00–1.35 mmol/L; Table 2). The flow rate of 10% calcium gluconate was increased by 10% if the iCa^2+^I was <1.0 mmol/L, or was reduced by 10% when iCa^2+^I was >1.35 mmol/L. The flow rate of 5% sodium bicarbonate was adjusted to maintain the relatively normal concentration of sodium ions and bicarbonate root.

**Table 2:**
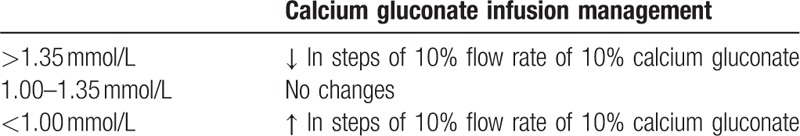
Management of calcium gluconate 10% infusion, dependent on systemic whole-blood ionized calcium levels (iCa^2+^I).

A blood gas analysis and test of electrolytes were conducted every 30 minutes after the start of the treatment, both in vivo and in vitro, until the anticoagulation targets of calcium were achieved, and then routinely every 4 to 6 hours to maintain target concentrations. Another anticoagulation protocol, or stopping the CHF, was conducted if the ratio of total calcium to ionic calcium (T/iCa^2+^) was >2.5, and hypocalcemia could not be corrected by administering calcium gluconate.^[12]^ Hemofiltration was ended for any of the following reasons: achievement of therapeutic goals; coagulation of the filter; transmembrane pressure continuously >200 mm Hg; or continuous treatment for 72 hours (to replace pipeline and filter).^[13]^

### 2.3 Observation indexes and significance

The following data were collected prospectively from 2015 onwards as part of clinical care for all patients on CRRT in our PICU: demographics, reason for PICU admission, Pediatric Index of Mortality (PIM) 2 score, reason for CRRT, laboratory data (blood gas analyses, calcium ions in vivo and in vitro, and total serum calcium in vivo), transmembrane pressure, and bleeding or clotting (Table 3).

**Table 3:**
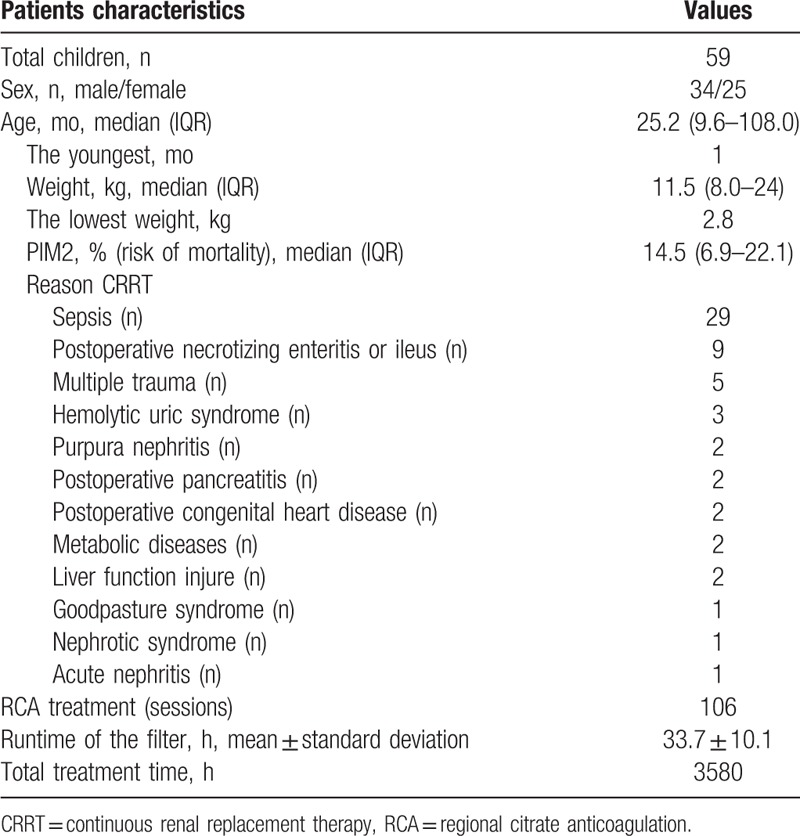
Patient characteristics.

### 2.4 Statistical analysis

Enumeration data were analyzed using the chi-squared test. Continuous variables consistent with normal distribution were reported as mean ± standard deviation and compared using the *t* test, or expressed as median (first and third quartile), and compared using the Mann–Whitney *U* test, as appropriate. Statistical analyses were performed with IBM SPSS version 21 software. A *P*-value < .05 was considered statistically significant.

## 3 Results

The study population included 59 pediatric patients with 106 admissions. The median age was 25.2 months (9.6–108.0 months) (range, 1.0–183.0 months), and the median weight was 11.5 kg (8.0–24.0 kg) (range, 2.8–50.0 kg). The RCA treatment duration was 33.7 ± 10.1 hours (range, 11.0–72.0 hours). The total treatment time was 3580 hours.

We retrospectively collected data from 637 sessions of iCa^2+^ in vivo and in vitro. During treatment, 563 (88.4%) sessions of iCa^2+^ in vitro achieved the target (0.25–0.35 mmol/L), with 26 (4.1%) sessions achieving <0.25 mmol/L, and 48 (7.5%) sessions achieving >0.35 mmol/L. In addition, there were 608 (95.4%) sessions where iCa^2+^ in vivo achieved the target (1.0–1.35 mmol/L), with 19 (3.0%) sessions achieving <0.9 mmol/L, and 10 (1.6%) sessions achieving >1.35 mmol/L.

### 3.1 Electrolytes, acid, and alkali before and after treatments

There were 637 sessions for which data were respectively collected, including Na^+^, iCa^2+^, and HCO_3_^−^ concentrations, pH value, T/iCa^2+^ ratio, and iCa^2+^ in vivo before and after treatment. After treatment, the average concentrations of iCa^2+^, T/iCa^2+^, Na^+^, and HCO_3_^−^, and pH were all significantly higher than before treatment (Table 4). Only 8 (7.5%) sessions with transmembrane pressure >200 mm Hg and 3 (2.8%) sessions with clotted filters were recorded. There were only 2 sessions with T/iCa^2+^ > 2.5, indicating citrate accumulation. There was 1 session with iCa^2+^ < 0.9 mmol/L, 2 sessions with iCa^2+^ > 1.35 mmol/L, and no session of sodium disorder after treatment. There were 2 sessions with iCa^2+^ < 0.9 mmol/L, 8 sessions with ionic sodium >150 mmol/L, and no sessions of iCa^2+^ > 1.35 mmol/L, T/iCa^2+^ > 2.5, or ionic sodium >150 mmol/L before treatment.

**Table 4:**
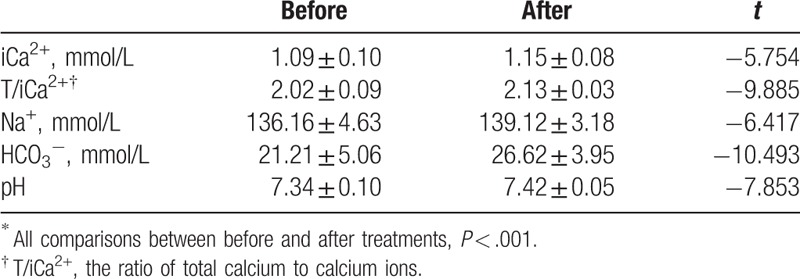
Electrolytes, acid, and alkali before and after 106 treatments^∗^.

### 3.2 Patients with sodium or calcium disorders, or acid–base imbalance

There were fewer cases of acidemia and more cases of alkalemia after treatment compared to before treatment (Table 5).

**Table 5:**
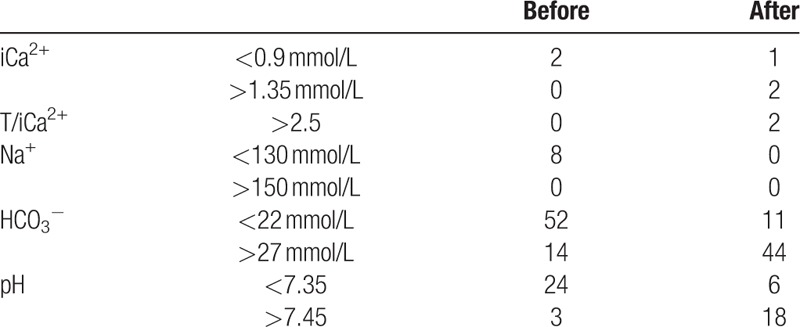
Number of patients with sodium or calcium disorders and acid–base imbalance before and after 106 treatments.

## 4 Discussion

CRRT has been widely used to treat critically ill children. As an extracorporeal circulation, CRRT needs anticoagulation, appropriately. Currently, RCA has been increasingly used to treat children due to its superior anticoagulant characteristics in vitro, but less so in vivo. Traditionally, calcium-free solutions are primarily used in RCA. There are several studies in adults that used calcium-containing solutions in RCA treatment,^[14,15]^ but there are no reports on its use in children. According to the results of a worldwide practice survey, CHF is one of the most commonly used modalities in CRRT, especially for patients with hemodynamic instability.^[16]^ To our knowledge, our study is the first to describe the application of RCA-CHF in children with a solution containing calcium. Based on our results, we conclude that RCA-CHF with a calcium-containing substitute is safe and effective for treating pediatric patients. RCA-CHF with a calcium substitute can be used to overcome pain when closely monitored, especially with regards to the calcium test site in vitro and the adjustment of citric acid or substitute in special cases.

In summary, our results showed that RCA with a substitute containing calcium could be safely used for CHF in children. To achieve the target concentration (0.25–0.35 mmol/L), we had to use more sodium citrate to chelate the extra calcium ions in the substitute containing calcium. The actual flow of sodium citrate (mL/hour) was related to the blood flow rate, as determined by the recommended formula: 1.8 × blood flow rate (mL/kg/minute). In this study, the concentration of sodium citrate in vitro was higher (4 mmol/L) than that reported in other studies using calcium-free substitutes (2.5–3.5 mmol/L).^[6,8,11]^ Additionally, there were only 2 sessions (1.9%) with T/iCa^2+^ > 2.5, which usually indicated citrate accumulation.^[17–19]^ This suggested that most children could tolerate a sodium citrate concentration of 4 mmol/L in the extracorporeal circulation. In addition, there were very few calcium disturbances, with only 1 session of iCa^2+^ < 0.9 mmol/L and 2 sessions of iCa^2+^ > 1.35 mmol/L. There were less cases of acidemia and more cases of alkalemia after treatment compared to before, with HCO_3_^−^ < 22 mmol/L (11 cases vs 52 cases), pH < 7.35 (6 cases vs 24 cases), HCO_3_^−^ > 27 mmol/L (44 cases vs 14 cases), and pH > 7.45 (18 cases vs 3 cases). These results may be due to several factors. First, severe illness or acute renal failure of the patients was easily complicated by acidosis. Secondly, the dose of sodium bicarbonate may need to be further reduced, especially in cases of alkalemia. Although the average iCa^2+^, T/iCa^2+^, Na^+^, HCO_3_^−^ concentrations, and pH were all significantly higher after treatment compared to before treatment, the average was still within or near the normal range. This indicates that it was safe to use RCA with a substitute containing calcium for CHF in children within 32.4 ± 11.6 hours.

We performed this study to clarify that a substitute containing calcium could effectively maintain the anticoagulant state of the extracorporeal circulation, without affecting total coagulation. In this study, there were 106 filters with a total time of 3450 hours. Moreover, although the average treatment time (33.8 ± 10.1 hours) was shorter compared to other studies (39.8 ± 4.8 hours),^[5–10]^ we had only 8 sessions (7.5%) with a transmembrane pressure >200 mm Hg and 3 sessions (2.8%) of clotted filters. This suggests that the filters may not meet the limit of the filter life, and the relatively short turnaround time might be more related to early achievement of the treatment goal.

In total, 637 pairs of calcium ions were collected during the treatment, including 563 (88.4%) in vitro that achieved the target (0.25–0.35 mmol/L) and 608 (95.4%) in vivo that achieved the target (1.00–1.35 mmol/L). Therefore, this study showed that RCA-CHF with a calcium substitute is safe and effective in children.

### 4.1 Site of monitoring calcium in vitro

When using a calcium-free substitute, the postfilter is always placed in the blood collecting location (point B in Fig. 1) to monitor the calcium concentration in vitro. However, when using a substitute containing calcium, the calcium concentration at point A will be higher than that at point B. Early in our attempts with this technique, we adjusted the flow of sodium citrate according to the calcium concentration at point B in 1 patient. With progressively increasing the transmembrane pressure, we found that the calcium concentration differed at points A and B (0.45 vs 0.21 mmol/L). Thus, the calcium concentration should be monitored in vitro at point A when using a substitute containing calcium.

### 4.2 Adjustment of citrate or displacement fluid

The flow of sodium citrate and substitute fluid needs to be adjusted in a timely manner according to the blood flow, which varies greatly when using CRRT with RCA for children. In this study, we gained experience from patients with high calcium concentrations in vitro. One patient with sepsis (weight 43.5 g) had the following initial flow rate parameters: blood 100 mL/minute; 4% sodium citrate 180 mL/hour; filtered solution 1.5 L/hour; and 10% calcium gluconate 10 mL/hour. Thirty minutes after beginning treatment, the calcium concentration in vitro was 0.55 mmol/L, which finally dropped to <0.35 mmol/L until the flow of 4% sodium citrate rose to 230 mL/hour. The filter duration was only 12 hours, perhaps because the in vitro calcium concentration at >0.35 mmol/L lasted for 6 hours. In this study, the practical flow rate of 4% sodium citrate was higher compared to other studies. This might be because there was more calcium due to ∼0.8 L HBS (the ideal weight of blood flow 100 mL/minute was 20 kg; the ideal flow rate of the filtered solution of 20 kg was 0.7 L). Therefore, we had to either reduce the flow rate of the filtered solution or increase the flow rate of the 10% calcium gluconate.

There has been no consensus regarding RCA-CHF in children with a substitute containing calcium. The present study is limited by a small sample size, leading to low statistical power in the results. In addition, the results were not adjusted for multiple confounders such as life-style, age, and environmental factors. To confirm our results, a larger patient population should be included in future studies.

Based on our findings, RCA-CHF with a substitute containing calcium can be safely and effectively used in children with close monitoring. In particular, the calcium test site in vitro and the adjustment of citrate should be given close attention.

## Author contributions

**Conceptualization:** Fang Zhou.

**Data curation:** Hongxing Dang.

**Methodology:** Ke Bai.

**Supervision:** Chengjun Liu.

**Writing – original draft:** Ke Bai.

**Writing – review & editing:** Feng Xu.
